# The Role of Root and Shoot Structures in CH_4_ Transport and Release in Wetland Plants

**DOI:** 10.3390/plants15071049

**Published:** 2026-03-29

**Authors:** Mengyu Ge, Yang Qiu

**Affiliations:** College of Ecology and Environment, Nanjing Forestry University, Nanjing 210037, China; 25yqiu@njfu.edu.cn

**Keywords:** methane emission, wetland plants, morphological barriers, root, shoot

## Abstract

Plant-mediated CH_4_ transport can enhance ecosystem CH_4_ emission by transporting soil-produced CH_4_. This pathway can exceed diffusion and ebullition as the dominant CH_4_ emission route. However, limited studies have investigated the morphological and anatomical factors influencing CH_4_ transport in plants. Through a series of manipulative experiments on the shoots and roots, this study examines the role of root and shoot structures in CH_4_ transport and release in six widespread wetland species: *Carex rostrata* Stokes, *Carex lasiocarpa* Ehrh., *Carex aquatilis* Wahlenb., *Iris pseudacorus* L., *Juncus effusus* L., and *Alocasia odora* (Lodd.) Spach. CH_4_ flux from all investigated species dropped significantly after clipping fine roots, while it did not change significantly after removing coarse roots. Shoot clipping and sealing significantly decreased CH_4_ flux from the investigated *Carex* species, but not from the other species. Our results demonstrate the important role of fine roots in controlling CH_4_ flux, whereas coarse roots play a minor role. Leaf blades are the major release site of CH_4_ from *Carex* species, while micropores at the shoot base are the primary release site of CH_4_ from the other species. Our study suggests that integrating plant-specific anatomical and morphological characteristics into global methane models is crucial to better predict and mitigate climate change impacts.

## 1. Introduction

Methane (CH_4_) is a powerful greenhouse gas due to its high global warming potential, efficient absorption of infrared radiation, and role in positive feedback mechanisms [1]. Despite covering only about 5% of the global land surface [2], wetlands contribute approximately 20–40% of the total global methane emissions [3]. CH_4_ emissions from wetlands depend on various factors, including temperature, water-table level (WTL), soil organic matter, soil redox potential (Eh), microbial community, and plant species [4,5,6,7,8,9]. Among these factors, plants play a crucial role as they influence CH_4_ emissions in multiple ways and the composition of plant communities has been found to be a better predictor of ecosystem CH_4_ flux than environmental parameters [10]. Plants can affect CH_4_ production by altering soil conditions and the availability of substrates for methanogens, known as the ‘substrate effect’ [11]. To adapt to anoxic environments, some wetland plants develop specialized structures called aerenchyma, which allow them to transport oxygen (O_2_) from the atmosphere to their roots [12]. The O_2_ can then diffuse into the surrounding soil, creating microaerophilic (low O_2_) conditions that favor methanotrophic activity, thus enhancing CH_4_ oxidation near the roots [13]. Conversely, soil-produced CH_4_ can be transported through the aerenchyma from the soil to the atmosphere, bypassing the aerobic zone where CH_4_ oxidation could occur, known as ‘conduit effect’ [14].

The conduit effect has been found to overshadow the influence of plants on CH_4_ production and oxidation within the ecosystem [15]. Specifically, the presence of plants can significantly increase overall ecosystem CH_4_ levels while depleting porewater CH_4_ concentrations [9]. Moreover, a substantial increase in ecosystem CH_4_ flux was observed when tubes were inserted into soils to mimic the conduit effect, excluding the substrate effect [16,17]. Similarly, the “ defoliation,” which involves the removal of foliage while keeping the stem above the soil/water surface, thereby isolating the conduit effect from the integrated effects of plants on CH_4_ production and oxidation, did not significantly affect CH_4_ emissions [18,19].

The rate of gas diffusion from plants to the atmosphere is lower than predicted based on the partial pressure of CH_4_ surrounding the roots, suggesting additional controlling factors [20,21]. These factors include resistance to CH_4_ transport from the rhizosphere into the roots, through the root-shoot interface, and from the plant to the atmosphere [22]. These factors have been widely studied in rice, and it is clear that either the rhizosphere–root interface or root–shoot interface restrain the transport [23,24,25]. In contrast, the rate-limiting interface for transport is unclear for most other wetland plant species. Among them, *Carex* spp. is mostly studied but with contradictory conclusion. Morrissey et al. [26] and Schimel [10] concluded that leaves and stomata is the rate-limiting step for CH_4_ transport in *Carex aquatilis*, whereas Kutzbach et al. [27] found that it is mainly restricted by the dense root exodermes.

However, to estimate the effects of aerenchyma plants on ecosystem CH_4_ emissions, identifying the anatomical and morphological factors limiting the transport is crucial [28,29]. Furthermore, plants vary significantly in their CH_4_ transport capacity, even within the same genus or plant functional types [14,30,31]. Grouping plants based on functional type could bias estimations of methane transport [9]. An alternative method might be investigating the traits restricting CH_4_ emissions across a wider range of species, especially those that are widely distributed globally and significantly affect CH_4_ emissions. Therefore, we conducted a series of manipulations on the shoots and roots of six widespread wetland plants (*Carex rostrata*, *Carex lasiocarpa*, *Carex aquatilis*, *Iris pseudacorus*, *Juncus effusus*, and *Alocasia odora*) to identify the specific sites restricting CH_4_ transport.

## 2. Results

### 2.1. CH_4_ Flux from Intact Plants

The mean CH_4_ flux from *Carex rostrata*, *Carex lasiocarpa,* and *Carex aquatilis* was 0.10 ± 0.46, 1.73 ± 0.55, and 0.99 ± 0.36 µmol CH_4_ h^−1^ plant^−1^, respectively. In comparison, the mean CH_4_ flux from *Iris pseudacorus*, *Alocasia odora*, and *Juncus effusus* was higher. *J. effusus* showed the highest CH_4_ flux, with a value of 7.35 ± 3.1 µmol CH_4_ h^−1^ plant^−1^, around 1.5 and 3 times higher than *I. pseudacorus* and *A. odora*, respectively. When expressed on a biomass basis, the overall interspecific pattern remained similar. The biomass-normalized CH_4_ fluxes of *C. lasiocarpa* and *C. aquatilis* were comparable, at 0.24 ± 0.08 and 0.23 ± 0.09 µmol CH_4_ g^−1^ DW h^−1^, respectively, and were approximately 10 times higher than that of *C. rostrata*. The biomass-normalized CH_4_ flux of *J. effusus* was 4.9 ± 2.07 µmol CH_4_ g^−1^ DW h^−1^, which was approximately 14 and 20 times higher than those of *I. pseudacorus* and *A. odora*, respectively.

The CH_4_ flux from *C. rostrata* and *C. lasiocarpa* was significantly correlated with shoot surface area (SA) and shoot dry biomass (DB) and fine root DB (both *p* < 0.001, Table 1). Additionally, the CH_4_ flux from *C. lasiocarpa* was also related to shoot cross-sectional area (CSA, *p* < 0.05). In contrast, the CH_4_ flux from *C. aquatilis* showed a different pattern, being significantly correlated only with the DB of fine roots (*p* < 0.001), while shoot SA and shoot CSA did not show significant correlations. For CH_4_ flux from *I. pseudacorus*, *A. odora*, and *J. effusus*, it was correlated with shoot SA, CSA as well as fine root DB (all *p* < 0.05), but not with coarse root DB. For all investigated species, fine root DB was correlated with shoot DB and SA.

### 2.2. Effects of Roots Manipulations on CH_4_ Flux

**Figure 1:**
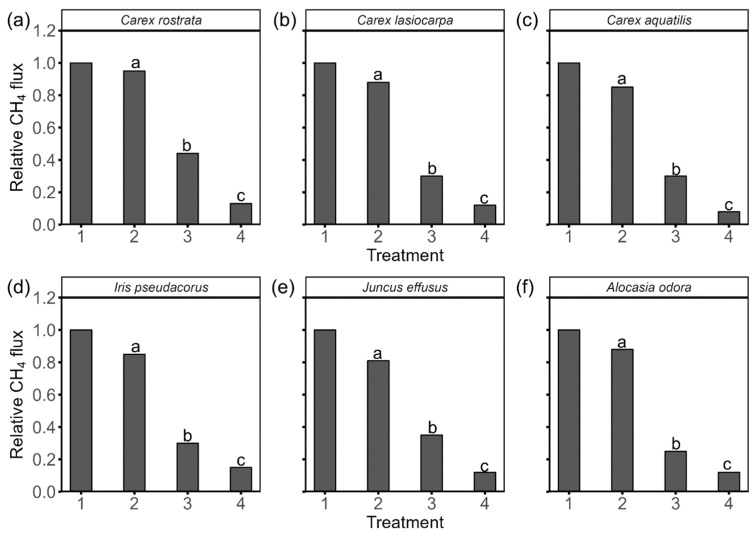
Relative CH_4_ flux from six plant species—*Carex rostrata* (**a**), *Carex lasiocarpa* (**b**), *Carex aquatilis* (**c**), *Iris pseudacorus* (**d**), *Juncus effusus* (**e**), and *Alocasia odora* (**f**)—under four different root treatments: 1, intact plant; 2, removal of all coarse roots; 3, cut fine roots at 150 mm; 4, cut fine roots at 50 mm. Letters denote statistically significant differences (*p* < 0.05) between the treatments.

CH_4_ flux from *C. rostrata* dropped by only 5% after removing coarse roots, while it decreased by 12% and 15% in *C. lasiocarpa* and *C. aquatilis*, respectively. Removing fine roots had a greater impact on CH_4_ flux for all investigated *Carex* species. While removing the distal 30 mm of the fine roots did not affect CH_4_ flux, cutting fine roots to a maximum length of 150 mm significantly decreased CH_4_ flux (Figure 1). When fine roots were cut to a maximum length of 50 mm, CH_4_ flux from *C. rostrata*, *C. lasiocarpa*, and *C. aquatilis* decreased by 87%, 88%, and 92%, respectively.

Similarly, removing coarse roots did not significantly decrease CH_4_ flux from *I. pseudacorus*, *A. odora*, and *J. effusus*, with reductions of 15%, 12%, and 19%, respectively (Figure 1). As with the *Carex* species, CH_4_ flux from these three plants was more affected by removing fine roots than by removing coarse roots. After cutting fine roots to a maximum length of 150 mm, CH_4_ flux from *I. pseudacorus*, *A. odora*, and *J. effusus* decreased by 70%, 75%, and 65%, respectively. Further reductions of 15%, 13%, and 23% were observed when fine roots were cut to a maximum length of 50 mm.

### 2.3. Effects of Shoot Manipulation on CH_4_ Flux

**Figure 2:**
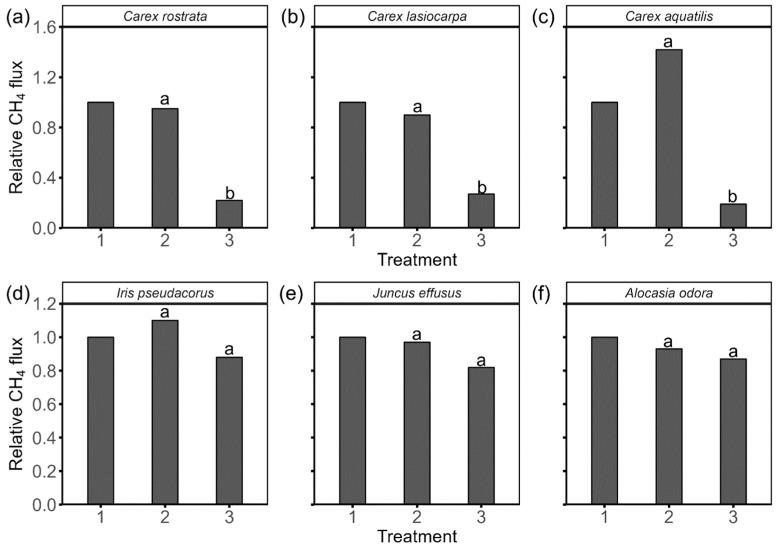
Relative CH_4_ flux from six plant species—*Carex rostrata* (**a**), *Carex lasiocarpa* (**b**), *Carex aquatilis* (**c**), *Iris pseudacorus* (**d**), *Juncus effusus* (**e**), and *Alocasia odora* (**f**)—under three different shoot treatments: 1, intact plant; 2, all shoots cut at 50 mm height; 3, seal the cutting ends. Letters denote statistically significant differences (*p* < 0.05).

Shoot manipulations had different effects on *C. rostrata*, *C. lasiocarpa*, and *C. aquatilis*. After clipping the shoots (main stem and plant above 10 cm from root), CH_4_ flux from *C. rostrata* and *C. lasiocarpa* did not change significantly. However, CH_4_ flux from *C. aquatilis* increased significantly, with an increase of 42% (Figure 2). The subsequent sealing led to a significant decrease of CH_4_ from all investigated *Carex*, with a relative CH_4_ flux of 22%, 27%, and 19% for *C. rostrata*, *C. lasiocarpa*, and *C. aquatilis*, respectively. As for CH_4_ flux from *I. pseudacorus*, *A. odora*, and *J. effusus*, it did not change significantly after shoot clipping and the further sealing.

## 3. Discussion

### 3.1. Fine Roots Control CH_4_ Flux from All Investigated Species

CH_4_ flux from all investigated plant species dropped significantly after removing fine roots (Figure 1), suggesting the important role of fine roots in regulating CH_4_ flux. Fine roots, with numerous lateral branches and root hairs, provide a large contact area with rhizosphere, facilitating effective gas and nutrient uptake [32,33]. Their high permeability allows gases to diffuse readily into the plant [34], while the well-developed aerenchyma in fine roots creates low-resistance pathways for internal gas movement [35]. Consistent with our findings, Yadav et al. [36] emphasized in their review that fine root traits—particularly aerenchyma development, root porosity, and radial oxygen loss—are critical determinants of plant-mediated CH_4_ transport in wetland plants. Our findings are consistent with Henneberg et al. [28] who concluded that removing fine roots significantly reduces CH_4_ flux.

In contrast, removing coarse roots did not significantly affect CH_4_ flux in any of the investigated plant species (Figure 1), highlighting the minimal role of coarse roots in CH_4_ regulation. Coarse roots have a thicker outer layer, often suberized or lignified, which serves as a barrier to radial O_2_ loss (ROL) [28,37]. This adaptation preserves O_2_ for internal root processes and enhances the plant’s survival in low O_2_ conditions [38]. Consequently, the primary functions of coarse roots are structural support and nutrient transport, rather than gas exchange [39].

However, our results do not exclude the possibility that coarse roots may still influence internal CH_4_ transport capacity. Although removing coarse roots did not significantly reduce CH_4_ flux in this study, this manipulation mainly tested whether coarse roots acted as a major entry point or external resistance to CH_4_ inflow. It did not directly examine whether internal anatomical features of coarse roots, such as aerenchyma development or diaphragms at the nodes, might influence gas transport efficiency. We therefore propose this as a hypothesis that warrants further anatomical and physiological investigation.

### 3.2. CH_4_ Is Mainly Released from the Leaf Blades of the Investigated Carex Species

The significant drop in CH_4_ flux from *Carex rostrata*, *Carex lasiocarpa*, and *Carex aquatilis* after shoot clipping and cutting-end sealing (Figure 2) suggests that most of CH_4_ might be released from leaf blades, not the shoot base. No significant changes in CH_4_ flux from *C. rostrata* and *C. lasiocarpa* after shoot clipping, suggest that CH_4_ release is not restricted by leaves and stomata. This is supported by the positive correlation between CH_4_ flux and surface area and shoot dry biomass (Table 1), as these factors determine the total surface area available for gas exchange and the plant’s overall capacity for CH_4_ production and transport. Our results align with Ge et al. [40], who did not observe significant changes in CH_4_ flux from *C. rostrata* after clipping leaf blades, and match Hu et al. [41], who reported that shoot clipping did not significantly alter CH_4_ flux from a *Carex cinerascens*-dominated meadow. *C. rostrata* and *C. lasiocarpa* often grow in wetter environments such as marshes, fens, and bogs, where water availability is high and humidity levels are generally elevated [9,42]. In such wet environments, they can afford to have larger and more stomata as they do not face significant water loss risk, allowing them to maximize gas exchange and photosynthetic efficiency [43,44].

In contrast, CH_4_ flux from *C. aquatilis* increased significantly after shoot clipping (Figure 2), suggesting that leaves and stomata might offer strong resistance to CH_4_ release. This might explain the poor correlation between shoot biomass and CH_4_ flux (Table 1), as even if shoot biomass increases, CH_4_ emission could be restricted by stomatal conductance. Our results are in line with Morrissey et al. [28] and Schimel [10], who highlighted the importance of stomatal control on CH_4_ release from *C. aquatilis* under field conditions. *C. aquatilis* is more commonly found in slightly drier habitats compared to the other two *Carex* species [45,46], leading to different adaptations in water use efficiency and gas exchange. Ge et al. [47] also found species-specific CH_4_ transport mechanisms, with root exodermis limiting transport in some species and leaf anatomy limiting it in others. In slightly drier environments, plants like *C. aquatilis* might develop fewer and smaller stomata to reduce water loss through transpiration, optimizing their stomatal features to balance gas exchange and water conservation [48,49].

However, the alternative explanation for the observed flux increase after clipping could be a physical “pressure release” effect; severing the shoot may instantly disrupt the tension within the plant’s gas lacunae, allowing for CH_4_ that was previously held in the roots to be rapidly drawn up and released. Further studies combining clipping experiments with simultaneous measurements of stomatal behaviour and internal gas pressure are needed to confirm the underlying mechanism.

Additionally, CH_4_ flux from *C. lasiocarpa* showed a significant correlation with shoot cross-sectional area (CSA, Table 1). A larger CSA likely allows for more efficient internal gas movement, enhancing CH_4_ release. This correlation, not observed in *C. rostrata* and *C. aquatilis*, implies different internal anatomical structures that affect CH_4_ emissions even within the same family. These differences could be due to varying adaptations to specific environmental conditions, affecting how each species processes and releases CH_4_.

### 3.3. Micropores on the Stem Are Primary CH_4_ Release Sites for I. pseudacorus, A. odora, and J. effusus

We did not detect significant changes in CH_4_ flux from *I. pseudacorus*, *A. odora*, and *J. effusus* after shoot clipping and cutting-end unsealing (Figure 2). This finding indicates that the shoots likely provide minimal resistance to gas transport, similar to what has been reported for other wetland plants like *Oryza sativa*, *Pontederia cordata* and *Sagittaria lancifolia* [23,50]. The hollow stems of *I. pseudacorus* and *J. effusus*, along with the large, spongy petioles and leaves with extensive aerenchyma in *A. odora* [51,52,53], act as efficient conduits for internal gas movement. The shoot walls likely impose minimal resistance to CH_4_ escape. Consequently, the larger shoots with greater surface areas and cross-sectional dimensions can thus enhance gas exchange, leading to increased CH_4_ emissions (Table 1).

We did not observe a significant change in CH_4_ flux from *I. pseudacorus*, *A. odora*, and *J. effusus* after sealing the cutting ends (Figure 2), suggesting that most CH_4_ might be released from micropores in the shoot base or below. If most CH_4_ was released from the shoots, sealing the cutting ends would significantly decrease CH_4_, as observed in the species *Carex* (Figure 2). Our findings are consistent with Henneberg et al. [28], who reported that CH_4_ escaped from *J. effusus* at 5 cm from the shoot base or below, and other wetland plants, e.g., rice [54] and *Scheuchzeria palustris* [55].

Our results are consistent with the presence of well-developed aerenchyma tissues extending from the roots to the shoot base in *I. pseudacorus*, *A. odora*, and *J. effusus*, which provide efficient low-resistance pathways for gas transport from anaerobic soil environments to the atmosphere [56]. Micropores at the shoot base, closer to the soil, facilitate effective CH_4_ release by providing a constant, low-resistance pathway, independent of environmental factors that regulate stomatal opening and closing [37].

### 3.4. Modeling Implication

To improve process-based CH_4_ modelling performance, our results suggest several key improvements. First, it is crucial to integrate detailed fine root dynamics, including their extensive surface area, high permeability, and aerenchyma development, as these traits significantly influence CH_4_ uptake and transport. Models should also incorporate fine root growth, turnover, and spatial distribution in the rhizosphere to accurately simulate CH_4_ absorption conditions [3,57]. Second, the distinct functions of coarse roots should be modelled separately from fine roots, reflecting their limited role in gas exchange. This differentiation will enhance the model ability to predict methane emissions accurately in wetland ecosystems.

A practical pathway for implementing these improvements emerges from our finding that, for all investigated species, fine root dry biomass (DB) correlated strongly with shoot dry biomass (DB) and surface area (SA) (Table 1). Given the difficulty of obtaining fine root data due to their small size and complex underground structure [58,59], this correlation provides a valuable proxy. By using the more easily obtainable shoot DB and SA to estimate fine root DB, models can simplify data collection processes while still achieving accurate predictions of plant-mediated CH_4_ emissions.

Furthermore, current models typically consider only the bulk biomass or leaf area index of aerenchymatous plants when estimating ecosystem-scale CH_4_ emissions [60,61]. Our results highlight the importance of moving beyond bulk biomass to consider the distinct anatomical structures and release mechanisms influencing CH_4_ emissions across different plant species. Given the high plant diversity even within a single wetland site, incorporating all species individually is unrealistic. To address this issue, we propose classifying plants into functional groups based on their CH_4_ transport mechanisms and anatomical traits (e.g., “leaf-releasers” vs. “micropore-releasers”). By identifying key representative species within each functional group, models can more accurately capture the variability in CH_4_ emissions without needing to account for every individual species. This approach balances complexity and practicality, improving model performance while remaining feasible for large-scale ecosystem applications.

Taken together, our study demonstrates that incorporating these varied mechanisms—root, leaf, and micropore dynamics—into ecosystem CH_4_ modelling is crucial for enhancing the accuracy of CH_4_ flux predictions across different wetland plant species and environments. Integrating these plant-specific traits with environmental variables such as soil CH_4_ concentration, water table levels, and temperature [3,62] will allow for a more comprehensive and accurate representation of CH_4_ emissions in diverse wetland ecosystems.

## 4. Materials and Methods

### 4.1. Plant Material

Small seedlings of *Carex rostrata*, *Carex lasiocarpa*, *Carex aquatilis*, *Iris pseudacorus*, *Juncus effusus*, and *Alocasia odora* were acquired from commercial nurseries (Baihui Huamu, Nanjing, China). The seedlings, along with the surrounding soil, were transferred to the laboratory. The soil was carefully rinsed from the roots to avoid damage, and the seedlings were placed separately into 30 L plastic water tanks. We created a floating raft from polystyrene with holes to hold the plants, ensuring their roots hung freely into the water below. The water tank was filled with a nutrient solution formulated for hydroponic cultivation, containing 200 ppm of nitrogen, 50 ppm of phosphorus, 200 ppm of potassium, 50 ppm of calcium, and 25 ppm of magnesium, along with essential micronutrients such as iron (Fe), manganese (Mn), zinc (Zn), copper (Cu), and boron (B) in trace amounts. The pH level was maintained between 5.5 and 6.5, and the nutrient solution was renewed weekly.

The seedlings were placed in a growth chamber (Beiyin Technology Co., Ltd., Suzhou, China) under a 14:10 h light/dark cycle, a photosynthetic photon flux density of 150 µmol m^−2^ s^−1^, a constant relative humidity of 60%, and a day/night temperature regime of 18/16 °C, which reflects typical environmental conditions in temperate wetlands. The seedlings were grown in the growth chamber for around 3 months before the measurement to acclimate to the hydroponic conditions and nutrient solution, ensuring the development of aerenchyma and root systems that were crucial for accurate CH_4_ transport measurements. The seedlings were observed daily to track the growth process and health status. The dead roots were removed, and tussocks were divided as needed, resulting in individuals with 2 to 18 shoots per species.

### 4.2. CH_4_ Flux Measurements

**Figure 3:**
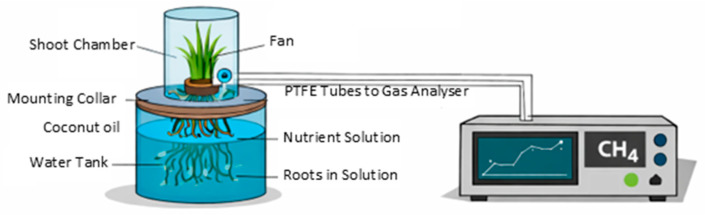
Schematic illustration of the laboratory setup.

To identify which plant parts restrict CH_4_ flux, we conducted a series of manipulation experiments on individual tussocks, each with 4–14 shoots and 7–40 roots. The experimental setup consisted of a 30 L plastic water tank (Chahua Modern Housewares Co., Ltd., Fuzhou, China) filled with nutrient solution, sealed with a lid fitted with a rubber seal (Figure 3). The lid had a hole with 10 mm diameter mounting collars to secure the plants, allowing the roots to extend into the nutrient solution while the shoots were enclosed in 1 L shoot chambers. Each shoot chamber was equipped with an electronic fan to circulate the air and included two ports for connecting gas inlet and outlet tubes.

Before flux measurements, we infused the nutrient solution in the tank with CH_4_ (Shanghai Wechem Chemical Co., Ltd., Shanghai, China) until it reached a concentration of around 1.16 mmol L^−1^, corresponding to about 75% saturation, which is at the higher end of concentrations typically observed in natural wetland soil water [63]. To ensure gas could only diffuse through the roots into the shoots, we used coconut oil (Wenchang Jiahua Co., Ltd., Wenchang, China) as a sealant to prevent gas diffusion, sealing the tussocks to the mounting platforms and preventing leakage into the shoot chamber. After these preparations, the tank was placed back into the growth chamber under the same environmental settings.

All CH_4_ flux measurements were conducted at approximately 12:00, corresponding to the middle of the light period (4 h after lights on), to minimize the influence of diurnal variation in stomatal conductance. When the flux measurement started, the shoot chamber was mounted, and air was continuously circulated between the closed shoot chamber and a gas analyser (LGR-UGGA, Los Gatos Research, Mountain View, CA, USA) using polytetrafluoroethylene (PTFE) tubes. Each measurement lasted 5 min.

We first measured the CH_4_ flux from intact plants, then subjected the plants to a series of manipulations targeting either the roots or shoots (Table 2). It should be mentioned that the aboveground parts of all investigated species were divided into the shoot and base. The shoot was defined as the part of the plant above the ground up to 5 cm in height, consisting mainly of leaf blades and leaf sheaths. The section below 5 cm was considered the base. Therefore, when all shoots were cut at a height of 100 mm (shoot manipulation 2), the changes in CH_4_ flux could reveal the resistance of the leaf blades to CH_4_ transport throughout the plant. By sealing the cutting ends (shoot manipulation 3), CH_4_ was expected to be released only from the shoot base, and thus the changes in CH_4_ flux before and after sealing could reveal the role of the shoot base in CH_4_ release. Since the plants could not be returned to their original state after manipulation, each plant was used for only one specific type of manipulation (either shoot or root). For each species, three tussocks were used for shoot manipulations (replicate = 3), and three different tussocks were used for root manipulations (replicate = 3).

After the flux measurements and manipulation experiments, all plant materials, including the tissues excised during the manipulations and the remaining plant parts, were retained for further morphological and biomass measurements. The materials were separated into shoots, coarse roots, and fine roots. Shoot surface area and cross-sectional area were determined using a digital scanner (PIXMA MG2580S, Canon Inc., Tokyo, Japan) and the IMAGEJ program (National Institutes of Health, Bethesda, MD, USA) [64]. The plant materials were then oven-dried at 60 °C until constant weight. Total dry biomass was calculated as the sum of the dry biomass of shoots, coarse roots, and fine roots, thereby representing the original total biomass of each intact plant prior to manipulation. For intact plants, CH_4_ flux was further normalized to total dry biomass and expressed on a biomass basis (µmol CH_4_ g^−1^ DW h^−1^). For the root and shoot manipulation experiments, relative CH_4_ flux was retained as the primary response variable because the treatments directly altered plant biomass and organ structure.

### 4.3. Statistical Analysis

The data analysis was conducted in R v3.6.1 (R Foundation for Statistical Computing, Vienna, Austria; https://www.r-project.org/) [65]. To ensure data quality, the dataset was initially examined through several steps: (i) outliers were detected using boxplots, (ii) the Shapiro–Wilk test was employed to assess normality, and (iii) Levene’s test was utilized to evaluate the homogeneity of variances among various subpopulations. For intact plants, CH_4_ flux was analysed both on a per-plant basis and on a biomass-normalized basis, with total dry biomass calculated as the sum of shoot, coarse root, and fine root dry biomass. Pearson correlation analysis was used to investigate the relationships between CH_4_ flux and plant morphological parameters, including shoot surface area, shoot cross-sectional area, shoot dry biomass, and fine and coarse root dry biomass. For the root and shoot manipulation experiments, differences in relative CH_4_ flux among sequential manipulations were analysed using analysis of variance (ANOVA) followed by Tukey’s post hoc test.

## 5. Conclusions

Our study highlights the importance of fine root dynamics, leaf blade involvement, and species-specific gas exchange mechanisms in plant-mediated CH_4_ transport. Through manipulative experiments, we show that fine roots are the predominant gateway for CH_4_ entry into plants, suggesting that root health, architecture, and soil–root interactions are critical for accurate CH_4_ flux estimates. In contrast, coarse roots appear to play a limited direct role in gas exchange due to their suberized or lignified barriers, serving primarily structural functions. For the *Carex* species, leaf blades were identified as the major CH_4_ release site, emphasizing the need to incorporate leaf dynamics and stomatal behaviour into models, especially for species growing in wetter environments with larger and more numerous stomata. Conversely, in *Iris pseudacorus*, *Alocasia odora*, and *Juncus effusus*, CH_4_ was primarily released through micropores at the shoot base, indicating a fundamentally different pathway that bypasses stomatal control. Together, these findings reveal distinct species-specific CH_4_ transport and release strategies. Further progress in understanding these mechanisms will require direct anatomical quantification, particularly of aerenchyma traits (e.g., fractional area) in roots and shoots, to provide stronger anatomical support for plant-mediated CH_4_ transport pathways.

## Figures and Tables

**Table 1:** Pairwise correlations between morphological parameters (shoot surface area, shoot cross-sectional area, shoot dry biomass, and fine and coarse root dry biomass) and CH_4_ flux from six widespread wetland plants (*Carex rostrata*, *Carex lasiocarpa*, *Carex aquatilis*, *Iris pseudacorus*, *Juncus effusus*, and *Alocasia odora*). Abbreviations: SA, surface area; CSA, cross-sectional area. Significance levels: *, *p* < 0.05; **, *p* < 0.005; ***, *p* < 0.0001.

***C. rostrata***	**CH_4_ Flux**	**Shoot SA**	**Shoot CSA**	**Shoot DB**	**Coarse Root DB**	***I. pseudacorus***	**CH_4_ Flux**	**Shoot SA**	**Shoot CSA**	**Shoot DB**	**Coarse Root DB**
Shoot SA	0.71 ***					Shoot SA	0.78 ***				
Shoot CSA	0.21	0.79 ***				Shoot CSA	0.35 *	0.70 ***			
Shoot DB	0.65 ***	0.89 ***	0.53 **			Shoot DB	0.65 ***	0.85 ***	0.50 **		
Coarse root DB	0.24	0.41 *	0.41 *	0.63 ***		Coarse root DB	0.20	0.45 **	0.32 *	0.55 **	
Fine root DB	0.82 ***	0.54 **	0.47 *	0.69 ***	0.19	Fine root DB	0.80 ***	0.60 **	0.45 *	0.70 ***	0.13
***C. lasiocarpa***	**CH_4_ flux**	**Shoot SA**	**Shoot CSA**	**Shoot DB**	**Coarse root DB**	***A. odora***	**CH_4_ flux**	**Shoot SA**	**Shoot CSA**	**Shoot DB**	**Coarse root DB**
Shoot SA	0.85 ***					Shoot SA	0.82 ***				
Shoot CSA	0.41 *	0.65 ***				Shoot CSA	0.40 *	0.72 ***			
Shoot DB	0.75 ***	0.91 ***	0.55 **			Shoot DB	0.68 ***	0.87 ***	0.52 **		
Coarse root DB	0.35	0.31	0.42 *	0.51 **		Coarse root DB	0.25	0.12	0.04	0.22	
Fine root DB	0.79 ***	0.66 **	0.49 *	0.71 ***	0.31	Fine root DB	0.85 ***	0.64 **	0.48 *	0.75 ***	0.33
***C. aquatilis***	**CH_4_ flux**	**Shoot SA**	**Shoot CSA**	**Shoot DB**	**Coarse root DB**	***J. effusus***	**CH_4_ flux**	**Shoot SA**	**Shoot CSA**	**Shoot DB**	**Coarse root DB**
Shoot SA	0.28					Shoot SA	0.81 ***				
Shoot CSA	0.19	0.68 ***				Shoot CSA	0.43 *	0.39 *			
Shoot DB	0.24	0.78 ***	0.27			Shoot DB	0.75 ***	0.77 ***	0.44 *		
Coarse root DB	0.17	0.35 *	0.11	0.39		Coarse root DB	0.14	0.03	0.12	0.17	
Fine root DB	0.74 ***	0.58 **	0.31	0.63 ***	0.42 *	Fine root DB	0.90 ***	0.67 ***	0.40 *	0.65 ***	0.03

**Table 2:** Summary of the sequential manipulation procedures used in the CH_4_ flux experiments. Root and shoot manipulations were conducted on different tussocks. Each step was performed sequentially on the same plant within each manipulation type.

(a) Root Manipulation		(b) Shoot Manipulation	
Step	Procedure	Step	Procedure
1	Intact plant	1	Intact plant
2	Remove all coarse roots	2	Cut all shoots to 100 mm height
3	Cut fine roots to 150 mm length	3	Seal the cut ends
4	Cut fine roots to 50 mm length		

## Data Availability

Data used in this study are available on request from the corresponding author.
